# Botulinum Toxin Therapy: A Comprehensive Review on Clinical and Pharmacological Insights

**DOI:** 10.3390/jcm14062021

**Published:** 2025-03-16

**Authors:** Nahla Ayoub

**Affiliations:** Department of Pharmacology and Toxicology, Faculty of Medicine, Umm Al-Qura University, Makkah 24375, Saudi Arabia; naayoub@uqu.edu.sa; Tel.: +966-532190083

**Keywords:** botulinum toxin, SNARE proteins, therapeutic applications, adverse effects, immunogenicity, novel serotypes

## Abstract

**Background:** Botulinum toxin (BoNT), produced by Clostridium botulinum, has transitioned from being a lethal neurotoxin to a versatile therapeutic agent. Its ability to inhibit neurotransmitter release by targeting Soluble N-ethylmaleimide-sensitive factor Attachment Protein Receptor (SNARE) proteins underpins its applications in treating conditions such as spasticity, dystonia, chronic pain, and overactive bladder. The clinical and pharmacological properties of BoNT have been extensively studied, with significant advancements in its therapeutic use, safety profile, and understanding of associated adverse effects. **Objective:** This comprehensive review aims to consolidate historical developments, molecular mechanisms, clinical applications, and challenges associated with BoNT, with a focus on expanding its therapeutic scope while ensuring safety and efficacy. **Method:** A narrative approach was used to analyze and synthesize insights from 155 references spanning experimental studies, clinical trials, and reviews. Key topics included BoNT’s historical milestones, mechanisms of action, therapeutic applications, and adverse events. **Findings:** BoNT demonstrates remarkable efficacy in a wide range of medical and cosmetic applications. In movement disorders such as dystonia and spasticity, it reduces muscle overactivity and improves functional outcomes. In chronic pain management, including migraines and neuropathic pain, BoNT significantly alleviates symptoms by modulating neurotransmitter activity. Cosmetic use for conditions like glabellar lines and hyperhidrosis highlights its precision and safety when administered appropriately. For conditions like strabismus and blepharospasm, BoNT effectively restores muscle control, reducing involuntary contractions. In urological applications, BoNT has proven to be an effective therapy for overactive bladder, offering significant symptom relief in refractory cases. However, concerns about long-distance effects, where the toxin may spread beyond the injection site to affect distant muscles or systems, have been reported in certain high-dose or sensitive populations. These findings emphasize the importance of dose optimization and patient-specific approaches. Adverse effects such as localized pain, hematoma, dysphagia, and systemic effects, particularly in high-risk groups, underscore the need for careful monitoring. The development of immunogenicity, leading to neutralizing antibodies, remains a challenge that impacts long-term therapeutic efficacy. Emerging research on novel serotypes, including BoNT/X, and innovations in delivery mechanisms, offer promising avenues to address current limitations. Advances in optimizing dosing regimens and refining injection techniques have also contributed to minimizing complications and improving outcomes across diverse patient populations. **Conclusions:** BoNT remains a cornerstone in neurology and cosmetic medicine, with its therapeutic potential still expanding. The balance between efficacy and safety, driven by innovations in formulation and application, underscores the importance of continued research. Future directions should focus on minimizing adverse effects, reducing immunogenicity, and exploring novel indications to further enhance its clinical utility.

## 1. Introduction

### 1.1. Botulinum Toxin History

Botulinum toxin (abbreviated either as BTX or BoNT) is produced by Clostridium botulinum, a Gram-positive anaerobic bacterium [1]. The clinical syndrome of botulism can occur following ingestion of contaminated food, from colonization of the infant gastrointestinal tract, or from a wound infection [1].

The German physician and poet Justinus Kerner (1786–1862) first developed the idea of a possible therapeutic use of botulinum toxin (BoNTs), which he called “sausage poison”. Sausage plays a prominent role in the story of the medical use of botulinum toxin. Sausage has been a delicacy for centuries, and, in the Byzantine era, blood sausage was commonly made by taking animal blood, fat, and organs, cooking them for varying amounts of time, and then stuffing them into “cleaned” animal stomach or intestine. Although the bacterial etiology of food poisoning was centuries away, the Byzantine emperor Leo VI embraced the association of blood sausage with food-related illness and later signed an edict that forbid the making and eating of blood sausage prepared in pig stomachs [2]. He first developed the idea of a possible therapeutic use of botulinum toxin, which he called “sausage poison”.

**Figure 1:**
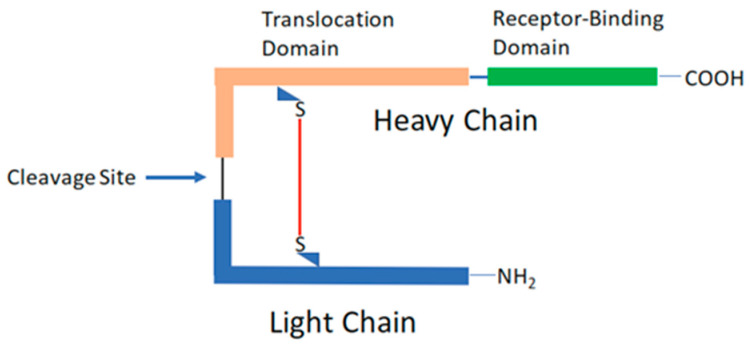
Botulinum toxin structure (schematic diagram) [22].

Further advancements into understanding the relationship between sausage and illness came during the Napoleonic War, which took place from 1795 to 1813. The war led to poor sanitary conditions in rural food production, and many deaths in Europe were associated with eating smoked blood sausages. These sausage-related deaths were studied, and, in the early 1800s, the Department of Internal Affairs of the Kingdom of Wurttemberg attributed this food poisoning to a substance they called prussic acid. However, it was the physician and poet, Dr. Justinus Andreas Christian Kerner (Figure 1), who made strong scientific inroads into our understanding of food poisoning, the role of botulism, and even the potential medical uses of botulinum toxin [3]. He described experiments, including those he performed on himself by eating small amounts of so-called sour sausage, and documented the signs and symptoms of botulism including vomiting and intestinal spasms, mydriasis, ptosis and strabismus, dysphagia, flaccid paralysis, and respiratory failure. He also noted that sausage poison develops under anaerobic conditions, interrupts motor signal transmission in the peripheral and autonomic nervous system, and is lethal in small doses. Incredibly, Kerner also proposed that the toxin could be used for therapeutic purposes. He hypothesized that this toxin could be used to lower sympathetic nervous system activity associated with movement disorders and decrease the hypersecretion of body fluids [4].

Around 1895, Emile Pierre-Marie van Ermengem, a bacteriologist from the University of Ghent, successfully isolated the bacterium responsible for producing botulinum toxin. This significant development was prompted by another outbreak of food poisoning, where the source was identified as spoiled ham, unlike the earlier incidents involving sour sausage. This work by van Ermengem marked a crucial step in understanding and identifying the cause of botulism [5].

The discovery of Clostridium botulinum, the bacterium responsible for botulism, was a pivotal moment in medical microbiology. It was first isolated and identified by Emile Pierre-Marie van Ermengem in 1895, following an outbreak of food poisoning in Belgium where several musicians fell ill and some died after consuming contaminated ham. Van Ermengem’s research led to the identification of the anaerobic bacterium, which he named Bacillus botulinum, derived from the Latin word for sausage, “botulus,” due to its initial association with meat products. This discovery was crucial in understanding the pathogenesis of botulism and paved the way for further research and eventual medical applications of botulinum toxin [6].

Following the early identification of botulinum serotypes by Burke in 1919 [7], the bacterial exotoxin was purified and crystallized in the 1920s, as documented by Snipe and Sommer [8]. This led to a deeper investigation into botulinum toxin’s mechanism of action. Edmunds and colleagues, in 1924 [9], revealed its curare-like effect on motor nerve endings, causing paralysis and notably impacting respiratory muscles, potentially leading to death [9]. Guyton’s team further detailed the toxin’s peripheral impact. By the late 1940s, it was established that the toxin obstructed neuromuscular transmission [9].

In the 1950s, Dr. Vernon Brooks discovered that injecting BoNT-A into an overactive muscle blocks the release of acetylcholine from motor nerve terminals. This paved the way for further research, and, in 1973, Dr. Alan B. Scott from the Smith-Kettlewell Eye Research Institute used BoNT-A in monkey experiments [10], later administering it to humans in 1980 to treat strabismus. BoNT-A, branded as BOTOX^®^, was approved by the US FDA in December 1989 for treating conditions such as strabismus, blepharospasm, and hemifacial spasm in patients under 12 years old [11].

Over time, the scope of BOTOX^®^’s applications expanded. On 21 December 2000, the US FDA approved BoNT-A for treating cervical dystonia [12]. The United Kingdom and Canada also approved BOTOX^®^ in 2001 for axillary hyperhidrosis, with Canada additionally allowing its use for focal muscle spasticity and cosmetic treatments for wrinkles. On 15 April 2002, the US FDA approved BOTOX^®^ Cosmetic for improving the appearance of frown lines between the eyebrows, known as glabellar lines. Later [12], in 2011, incobotulinumtoxinA (Xeomin) received approval for the same cosmetic purpose.

In July 2004, BOTOX^®^ gained FDA approval to treat severe underarm sweating, termed primary axillary hyperhidrosis, when topical treatments were ineffective [13]. BoNT-A’s acceptance for chronic pain management has also grown, though its FDA approval in this area remains limited to chronic migraines. The clinical application of BoNT-B has also been explored, with products like MyoBloc in the US and NeuroBloc in Europe. MyoBloc was FDA-approved on 8 December 2000, for cervical dystonia [14]. Research into BoNT-F’s use is ongoing, particularly for patients resistant to serotypes A and B. On 29 April 2009, abobotulinumtoxinA (Dysport) was approved by the FDA for cervical dystonia in adults. Further approvals include BOTOX^®^ in March 2010 for treating spasticity in adults recovering from strokes or traumatic brain injuries [15] and Xeomin^®^ in August 2010 for cervical dystonia and blepharospasm. By October 2010, BOTOX^®^ was approved for preventing chronic migraines [16]. Additional approvals for urinary incontinence treatment [17] and aesthetic applications, such as treating crow’s feet, followed. In 2015, Dysport^®^ and Xeomin^®^ were also approved for treating upper limb spasticity [18], and, in 2019, Jeuveau^®^ was introduced for reducing glabellar lines [19]. Additionally, the most substantial and lasting improvements were observed with injections of 1000 units of Dysport^®^, but this came with a higher incidence of adverse events. As a result, it is recommended to start with a lower dose of 500 units in patients with cervical dystonia, with the option to increase the dose in future sessions if clinically needed [20] Table 1.

### 1.2. The Structure of Botulinum Toxin

For the past half-century, significant advances have been made in elucidating the molecular mechanisms underpinning the activity of the botulinum toxin. Current understanding reveals that this toxin is composed of a heavy and a light chain linked by a solitary disulfide bond, with the heavy chain incorporating a binding and a translocation domain as illustrated in Figure 1. Upon attachment to a cell, the light chain undergoes cellular uptake, where it targets a specific set of proteins responsible for neurotransmitter release, as described by Rizo and Sudhof in 1998 [21,22]. Functioning as an endopeptidase, the light chain proceeds to sever the proteins that are crucial for the fusion of neurotransmitter vesicles to the internal cellular membrane, resulting in a cessation of neural transmission. In more recent developments, researchers have visualized the crystal structure of botulinum toxin [23] and pinpointed the toxin’s corresponding receptor [9].

**Figure 2:**
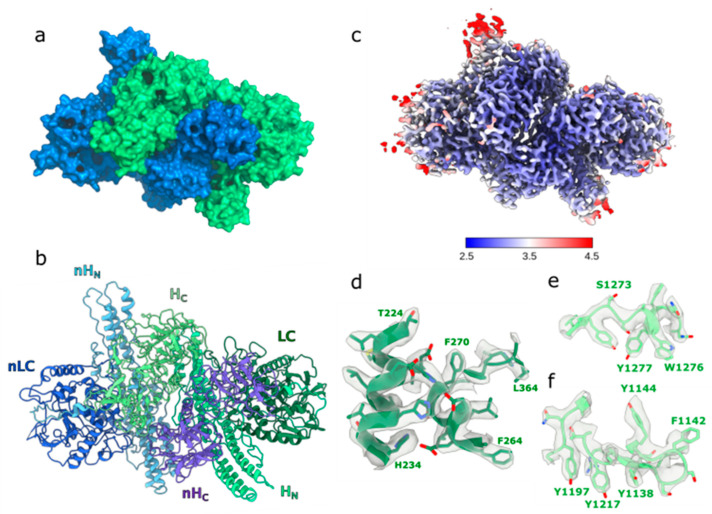
(**a**) Surface view of the BoNT-X (green)–NTNH (blue) complex. (**b**) Cartoon view of the different domains in each protein. BoNT-X domains are colored in green shades, and NTNH-X domains are colored in blue shades. (**c**) Local resolution estimates (Å) for the cryo- EM map of the M-PTC/X. (**d**) cryo-EM map around the LC active site. (**e**) cryo-EM map around the SxWY ganglioside-binding motif. (**f**) cryo-EM map around the HC patch rich in aromatic residues [24].

A recent study by Martínez-Carranza (2023) [24] reported that botulinum neurotoxins (BoNTs) are naturally co-expressed with a protective partner protein known as Non-Toxic Non-Hemagglutinin (NTNH), forming a 300 kDa complex that provides resistance against acidic and proteolytic degradation within the digestive system. The study also identified a novel serotype, BoNT/X, which possesses unique properties with potential therapeutic applications. Structural analysis using cryo-electron microscopy (cryo-EM) revealed that the BoNT/X-NTNH/X complex, resolved at 3.1 Å, maintains stability and resistance to proteases at both neutral and acidic pH levels, only disassembling under alkaline conditions. Despite BoNT-X exhibiting high catalytic activity and efficient translocation, its overall potency was found to be significantly low in both in vitro and in vivo studies when isolated with the aid of NTNH. This reduced activity is likely due to the weak binding affinity of its receptor-binding domain to gangliosides and exposed hydrophobic surfaces (Figure 2).

### 1.3. Botulinum Neurotoxin Mechanism of Action

BoNTs are produced by bacteria of the genus Clostridium though other bacteria of different classes and even phyla may harbor the gene encoding for BoNT-like proteins. They consist of two chains (L, 50 kDa and H, 100 kDa) linked by a single SS bridge [25,26]. They are produced in eight different serotypes (indicated by letters BoNT-A, BoNT-B, BoNT-C, BoNT-D, BoNT-E, BoNT-F, BoNT-G, and BoNT-X) [26]. The mechanism of action of botulinum neurotoxins (BoNTs) involves a series of precise steps five major steps (1) binding to cholinergic nerve terminals [27], (2) entry inside recycling synaptic vesicles (SV) [28,29,30,31], (3) the crossing of the vesicle membrane by the L domain by exploiting the pH gradient (acid inside) across the membrane [32], (4) the release of L in the cytosol by a reduction in the interchain disulphide bond [33,34,35], and (5) the cleavage of one or more of the three proteins that form the SNARE heterotrimeric complex that is essential for the fusion of synaptic vesicle with the presynaptic membrane, thus releasing their neurotransmitter content [26,35,36,37]; finally, this leads to the inhibition of neurotransmitter release in neurons, resulting in paralysis [36,38].

**Figure 3:**
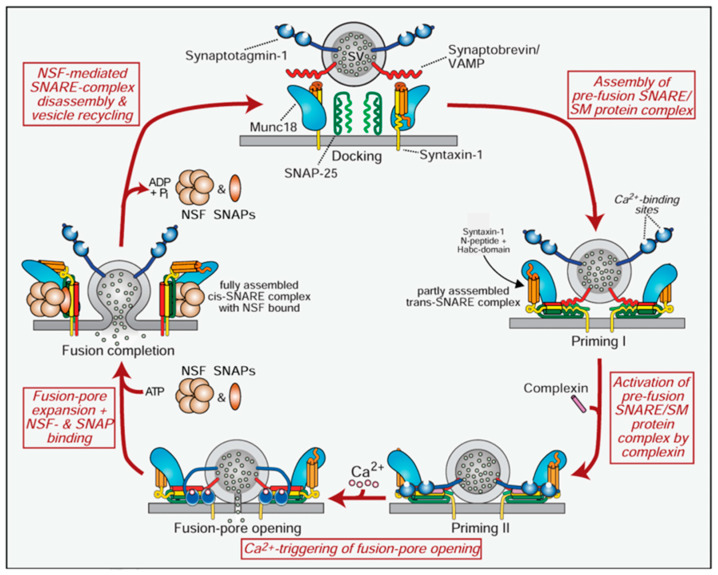
Schematic of the SNARE/SM protein cycle mediating fusion, and the role of synaptotagmin and complexin in Ca^2+^—triggering of fusion [40].

The process begins with the toxins binding to specific receptors on the surface of neurons. BoNTs utilize a dual-receptor system: first, they bind to gangliosides, a class of glycosphingolipids found on neuronal membranes, which helps them attach to the neuron surface. They then bind to a protein receptor, such as synaptotagmin or SV2, on the synaptic vesicle membranes. This dual binding ensures that the toxins are anchored securely to the neurons [27]. After binding, the toxins are internalized into the nerve terminals via receptor-mediated endocytosis. Within the acidic environment of the endosome, the heavy chain undergoes a conformational change that facilitates the translocation of the light chain into the cytosol of the neuron. Once in the cytosol, the light chain, which is a zinc-dependent protease, cleaves specific SNARE proteins. SNARE proteins are crucial for the fusion of synaptic vesicles with the plasma membrane, a process essential for the release of neurotransmitters. Botulinum neurotoxin (BoNT) exerts its effect by cleaving SNARE proteins, which are essential for neurotransmitter release. After entering neurons via endocytosis, the light chain of BoNT acts as a zinc-dependent protease, targeting specific SNARE proteins such as SNAP-25, VAMP/synaptobrevin, or syntaxin. This cleavage prevents synaptic vesicle fusion with the presynaptic membrane, leading to inhibited neurotransmitter release and muscle paralysis (Figure 3). This specific targeting and inhibition of neurotransmitter release make these neurotoxins both highly potent and medically valuable for conditions requiring muscle relaxation [26,39,40].

### 1.4. Duration of Action

The different durations of the action of botulinum neurotoxins likely addresses how various serotypes of BoNTs exhibit varying durations of their therapeutic effects due to differences in their molecular structures and interactions with target cells. Factors influencing the duration of action include the specific binding affinity of the toxin to its receptors, the rate of internalization and translocation into neurons, and the stability of the toxin within the cell. For example, BoNT-A is known for its longer duration of action, often lasting several months, which makes it particularly useful in clinical settings for treating conditions like muscle spasticity and chronic migraines. In contrast, BoNT-E typically has a shorter duration, which could be due to faster degradation or less stable binding within the neurons [36]. Additionally, the effectiveness and safety of BoNT-A treatment for cervical dystonia have been confirmed over a prolonged period, lasting up to 14 years [41].

### 1.5. Long-Distance Effects of Botulinum Neurotoxins

Botulinum neurotoxin type A (BoNT-A) is a metalloprotease known for producing a sustained but temporary blockade of neurotransmitter release from peripheral nerve terminals. However, it has been recognized that not all effects of BoNT-A can be solely attributed to its action at peripheral nerve terminals. Evidence suggests that BoNT-A may possess operating properties similar to those of the tetanus neurotoxin, enabling it to directly influence the central nervous system (CNS) [42]. Compelling evidence of BoNT-A1 retrotransport to the central nervous system (CNS) was provided by tracing the cleavage of SNAP-25 within the CNS neurons after the peripheral injection of the toxin, using an antibody very specific for the novel epitope generated by the BoNT-A1 cleavage of SNAP-25 [42,43]. BoNT-A1 retrograde transport can occur also via sensory neurons, as shown by the injection in the whisker pad, which induces the appearance of truncated SNAP-25 in the trigeminal nucleus caudalis [43]. Mazzocchio R, Caleo M.’s study [44] provided strong evidence of BoNT-A’s direct impact on the CNS, specifically in both the dorsal and ventral horns of the spinal cord in animals and humans after peripheral administration. The discovery of BoNT-A’s central action is expected to transform the future application of this neurotoxin in clinical practice. As the central effects of BoNT-A also appear to contribute to functional improvements, such as enhanced motor control in conditions like human spastic gait, there is a growing interest in developing new BoNT-A subtypes or derivatives. These derivatives would ideally have targeted, cell-specific effects within the CNS, allowing for a more comprehensive utilization of BoNT-A’s therapeutic potential [45] in the nervous system [46]. Moreover, it would aid the treatment of overactive bladder and voiding dysfunction in patients with central nervous system lesions [47].

## 2. Results

### 2.1. Therapeutic Uses of Botulinum Toxin in Clinical Practice

#### 2.1.1. Botulinum Toxin in Dystonia

The use of botulinum toxin type A (BoNT-A) in various forms of focal dystonias has been extensively researched and has shown significant effectiveness. In fact, botulinum toxin injections are the preferred treatment for cervical dystonia (also known as spasmodic torticollis) [48,49,50]. A Cochrane review determined that a single injection of BoNT-B is both effective and safe for treating cervical dystonia. This injection provides the greatest benefit to the highest proportion of patients in the shortest time, with its effectiveness confirmed in numerous double-blind, placebo-controlled studies [51].

- Cervical Dystonia

The effectiveness and safety of BoNT injections for managing various movement disorders, such as blepharospasm, hemifacial spasm, oromandibular dystonia, cervical dystonia, focal limb dystonias, laryngeal dystonia, tics, and essential tremor, continue to be studied [52].

In a double-blind, placebo-controlled trial conducted by Greene and colleagues, 55 patients who had not found relief from two prior medication trials were randomly assigned to receive either BoNT or placebo. The patients were monitored for 12 weeks. Following this, an open phase lasting four weeks was conducted, during which all patients received BoNT. By six weeks, 61% of patients showed improvement in head posture, and 39.5% reported a reduction in pain, with both outcomes significantly better (*p* < 0.05) compared to the control group. In the open phase, those who had previously received a placebo experienced a similar response. Overall, 74% of patients showed improvement by the study’s conclusion [53]. A study conducted by Brans and colleagues found that, among 64 patients with cervical dystonia, 84% experienced long-term improvements in impairment, disability, handicap, and the quality of life (QOL) [54]. Mezaki and colleagues shared their experience using a Japanese type A toxin to treat cervical and axial dystonias. Injections were administered at 28–30-day intervals to carefully selected muscles exhibiting increased activity, with a maximum dose of 300 units per session. Significant improvements in both subjective and objective assessments were only achieved after multiple injections [55]. In a study of 205 out of 232 patients with medically resistant cervical dystonia, tracked for 3 months to 4 years, a total of 1074 injections were administered over 505 visits. Of these patients, 71% showed significant improvement after one or more visits, with 76% of those experiencing pain reporting almost complete relief. Most patients noticed improvement within the first week after injection, though some had a delay of up to 8.5 weeks. The duration of maximum benefit varied, with some patients experiencing relief for up to 12.5 months, though the average was 11.2 weeks. Complications were minimal, affecting only 28% of patients, with mild dysphagia and neck weakness being the most common. Overall, the study concluded that botulinum toxin is a safe and effective treatment for the majority of patients with cervical dystonia [56]. Botulinum toxin (BoNT) injections have become the preferred treatment for focal dystonia, offering significant improvements when tailored to individual needs. Early studies lacked robust results due to poor design, but targeted injections can yield dramatic effects lasting 3 to 4 months. With experience, side effects like dysphagia and neck weakness are minimized. Although BoNT does not slow disease progression, early use has prevented long-term complications like contractures [57,58]. Regarding BoNT-B, a single injection was found to be both effective and safe in treating cervical dystonia. Long-term uncontrolled studies indicated that repeated injection cycles remained effective for the majority of patients [51,58,59,60]. Botulinum toxin B has proven effective for treating cervical dystonia at doses up to 10,000 U and is generally well tolerated, even in patients resistant to botulinum toxin A. Although the risk of developing secondary resistance to botulinum toxin B is unclear, it may be lower than with botulinum toxin A due to differences in manufacturing and preparation. Since botulinum toxin injections are the preferred treatment for cervical dystonia, botulinum toxin B should be considered a viable option in this context [61]. Comella et al. reported in 2005 that both botulinum toxin serotypes, BoNT-A and BoNT-B, provided similar benefits for patients with cervical dystonia after 4 weeks. However, BoNT-A had fewer side effects and a slightly longer duration of effectiveness in those who responded clinically [41,62]. Meanwhile, Comella et al. documented that there is limited evidence indicating that a single session of BoNT-A (onabotulinumtoxinA) and BoNT-B (rimabotulinumtoxin B) are equally effective and safe in treating certain forms of cervical dystonia in adults. However, BoNT-B seems to be associated with a higher likelihood of sore throat or dry mouth compared to BoNT-B. In general, the available single-treatment studies do not provide enough clinical evidence to recommend one type of botulinum toxin over the other [63]. Marques et al. in 2016 [51] found that a single treatment session with BoNT-B significantly and clinically reduces cervical dystonia symptoms, including severity, disability, and pain, and is well tolerated compared to a placebo. However, BoNT-B-treated patients have a higher risk of dry mouth and dysphagia. No randomized, placebo-controlled trials (RCTs) have assessed the safety and effectiveness of repeated BoNT-B injection cycles.

A recent study in 2021 reported that the introduction of low antigenicity BoNT formulations has enabled shorter intervals between injections, allowing for more flexible dosing and better management of extensive symptoms. Additionally, these formulations permit the use of higher doses without triggering immune resistance. High-affinity BoNT-B drugs may further enhance treatment by reducing antigenicity and lowering the risk of immune response, potentially allowing even higher doses to be used safely [10,50,64].

BoNT-A led to notable improvements in dystonic symptoms, psychiatric issues, pain, and disability, though it had no effect on sleep disorders. While motor and non-motor changes followed a similar timeline, motor improvements did not correspond with non-motor changes after treatment. Non-motor symptom changes following BoNT-A treatment are a complex process and are at least partially separate from improvements in motor symptoms [48,61,65,66,67]. Additionally, in 2021, Dressler et al. updated and improved consensus guidelines by (1) incorporating updated treatment algorithms, (2) analyzing real-world data from thousands of BoNT injections in dystonia and spasticity patients, (3) offering detailed dosing information, (4) adapting dosing for specific clinical conditions, and (5) providing concise reviews of the treated conditions alongside general BoNT therapy guidelines [68,69].

Despite BoNT’s effectiveness in treating cervical dystonia (CD), many patients experience poor outcomes and stop treatment. Factors contributing to suboptimal responses or treatment failure include incorrect muscle targeting, improper BoNT dosing, inadequate injection techniques, perceived lack of efficacy, and the development of neutralizing antibodies against the toxin [70]. The new COL-CAP classification for cervical dystonia may help improve muscle target identification. However, more precise information could be gained through kinematic or scintigraphic techniques, while using electromyographic or ultrasound guidance could enhance the accuracy of injections [70]. In 2024, Shukla reported that clinical effects of BoNT extend beyond peripheral muscle relaxation or adjusting muscle spindle input. Emerging evidence, though from smaller studies, indicates that BoNT modulates distant brain regions involved in dystonia. A single BoNT treatment has been shown to reduce motor excitability and enhance sensory processing [71]. Finally, a study in 2024 demonstrated a significant reduction in BoNT dosage after DBS surgery in patients with generalized dystonia, suggesting a potential combined effect of these treatments. It also highlighted that certain body areas, such as axial muscles, may respond more effectively to Deep Brain Stimulation (DBS), though further research is required to confirm this. Future prospective studies with well-defined treatment protocols and long-term analyses are needed to better understand the role of combining BoNT and DBS in managing generalized dystonia [72].

#### 2.1.2. Botulinum Toxin in Spasticity

Spasticity is characterized by an increase in muscle tone velocity. Intramuscular injections of botulinum toxin (BoNT) have been researched and proven beneficial for treating spasticity associated with conditions like the following:

- Multiple sclerosis (MS)

Botulinum toxin type A (BoNT-A) is an effective and safe treatment for managing spasticity, particularly in multiple sclerosis (MS) patients. BoNT-A injections reduce muscle tone and spasticity in both upper and lower limbs, improving outcomes related to mobility, hygiene, and pain. While spasticity reduction is significant, functional improvements such as enhanced mobility or hand function are dependent on patient-specific factors and realistic goal-setting. Long-term use of BoNT-A is generally well tolerated, with low discontinuation rates, though the effectiveness is often enhanced when combined with other therapies such as physical rehabilitation. Persistence in treatment is affected by factors like the interval between injections and patient-related variables, including cognitive function [73,74,75,76,77,78].

- Cerebral Palsy (CP)

Many studies highlight the effectiveness of botulinum toxin type A (BoNT-A) in reducing muscle spasticity in children with cerebral palsy. They agree that BoNT-A provides short-term improvements in muscle tone and the range of motion (ROM), but the functional gains, such as better mobility, tend to be temporary and diminish over time, usually lasting between 3 and 6 months. These studies caution against frequent use of BoNT-A due to concerns about muscle atrophy and long-term muscle changes, recommending a more cautious application of the treatment. In terms of research methodology, Tedroff et al. (2009) [79] conducted a prospective cohort study with a long-term follow-up of children receiving repeated injections, while Multani et al. (2019) [80] provided a review of multiple studies, analyzing the short- and long-term effects of BoNT-A and emphasizing the importance of functional and safety monitoring [79,80,81].

- Stroke

Regarding the effectiveness of botulinum toxin type A (BoNT-A) in reducing post-stroke spasticity, studies reported a significant improvement in muscle tone as measured by the Modified Ashworth Scale (MAS). The first study, a longitudinal cohort investigation [82], focuses on early BoNT-A injections within three months of stroke onset and finds that early intervention leads to more pronounced spasticity reduction at 4 and 12 weeks compared to later administration. The second study [83], a systematic review and meta-analysis, consolidates findings from multiple trials, confirming BoNT-A’s efficacy in reducing spasticity and improving functional outcomes like gait speed. Both papers recommend an early administration of BoNT-A for optimal management of spasticity, while also calling for further research to refine the timing of treatment and its impact on functional recovery [82,83,84,85].

- Traumatic brain injury (TBI)

The efficacy of botulinum toxin type A (BoNT-A) in managing spasticity across various neurological conditions is well documented in the literature, particularly in traumatic brain injury (TBI) and spinal cord injury (SCI). All the studies demonstrated significant improvements in muscle tone and spasticity, contributing to temporary but meaningful functional gains, such as improved gait and mobility. Despite the variations in treatment outcomes, BoNT-A’s safety profile remained favorable even at higher doses (over 600 units), with limited adverse effects reported. Regular injections, typically every 3 to 6 months, were found necessary to maintain the benefits of spasticity reduction. The research methods employed in these studies ranged from observational longitudinal designs and cohort studies to randomized controlled trials (RCTs) and cross-sectional surveys. These methodologies consistently relied on validated spasticity measures, such as the Modified Ashworth Scale (MAS), to assess treatment outcomes, affirming BoNT-A’s role in enhancing patients’ quality of life while also supporting its safe use in clinical practice [46,86,87,88,89].

#### 2.1.3. Botulinum Toxin in Pain Management

Through a review of literature and clinical trials, it was found that botulinum toxin is an effective treatment for lower back pain and myofascial pain syndrome, providing significant pain relief within three weeks, with effects lasting up to six months. The toxin works by relaxing muscles and inhibiting the release of pain-related neurotransmitters like substance P. While generally safe, the study notes that further randomized trials with larger populations and longer follow-ups are necessary to solidify its use as a cost-effective treatment [90].

A systematic review and meta-analysis evaluated the efficacy of botulinum toxin A (BoNT-A) in treating neuropathic pain (NP) by analyzing 17 randomized controlled trials (RCTs) conducted between 2005 and 2021. The study aimed to update the evidence on BoNT-A’s impact on pain reduction, the quality of life, sleep, and mental health outcomes, using data from sources like EMBASE, CINAHL, and ClinicalTrials.gov. The meta-analysis showed that BoNT-A significantly reduced pain, with a mean final Visual Analog Scale (VAS) reduction of 2.59 units compared to placebo. It also led to a 50% reduction in pain scores between baseline and final measurements, with a higher relative risk reduction compared to the placebo group. BoNT-A further decreased the frequency of neuralgia attacks, showing a notable improvement over the control group. However, no significant differences were observed between the groups for sleep, anxiety, depression, and quality of life measures. Despite these positive outcomes for pain reduction, the authors concluded that the available evidence is insufficient to support BoNT-A as a first-line treatment for NP due to limitations in the RCTs, such as small sample sizes and methodological biases. The study calls for more robust, well-designed trials to establish BoNT-A’s role as a primary treatment option for neuropathic pain [91].

The paper systematically reviews the efficacy of botulinum toxin type A (BoNT-A) in managing orofacial neuropathic pain (NP), analyzing six randomized controlled trials (RCTs) involving 795 patients and focusing on conditions like classical trigeminal neuralgia and post-herpetic neuralgia. The studies used various doses and methods of BoNT-A injections. Following PRISMA guidelines, the authors conducted a comprehensive search using databases such as MedLine, Web of Science, and the Cochrane Library, applying strict inclusion and exclusion criteria to select only RCTs assessing BoNT-A for orofacial NP. The results showed that BoNT-A significantly reduced pain levels compared to placebo, with pain relief occurring between 7 and 15 days post-injection and lasting up to 3 months. Additionally, five out of the six studies reported improvements in patients’ quality of life, though different scales were used for measurement. Reported side effects were generally mild and transient, including facial asymmetry, swelling, and injection site pain. The review concludes that BoNT-A is effective in reducing pain and enhancing the quality of life for patients with orofacial neuropathic pain, particularly for trigeminal neuralgia and post-herpetic neuralgia. However, it emphasizes the need for further research with larger sample sizes and improved study protocols to fully establish its clinical potential [92].

The paper reported the effects of BoNT-A on pain, somatosensory, and psychosocial features of patients with refractory masticatory myofascial pain in a randomized double-blind clinical trial and evaluated the efficacy of botulinum toxin-A (BoNT-A) on patients with refractory masticatory myofascial pain (MFP). The study involved 28 participants who were randomly assigned to receive either BoNT-A or saline solution injections in the masseter and anterior temporalis muscles. Outcomes measured included pain intensity (VAS), quantitative sensory testing (QST), conditioned pain modulation (CPM), and psychosocial status. The follow-up assessments were conducted at 1 and 6 months post-treatment.

The results showed that BoNT-A significantly reduced pain intensity compared to the saline group at both follow-ups. BoNT-A also increased the pressure pain threshold in the masseter muscle and showed a better CPM effect at the 1-month follow-up. Improvements were observed in psychosocial variables, including reductions in anxiety, depression, and sleep disturbances. The study concluded that BoNT-A had significant positive effects on pain, somatosensory function, and psychosocial factors in patients with refractory MFP, demonstrating its efficacy as a treatment option for this condition [88,92,93,94].

#### 2.1.4. Botulinum Toxin in Overactive Bladder (OAB)

Botulinum toxin A (Botox) has shown significant therapeutic benefits in treating refractory overactive bladder (OAB) and interstitial cystitis/bladder pain syndrome (IC/BPS), primarily by reducing bladder pain, frequency, and urgency. Clinical studies have demonstrated improved bladder capacity and the quality of life, particularly with repeated Botox injections, which extend the therapeutic duration compared to single doses. While 100 U doses are standard for balancing efficacy and side effects, adverse events such as urinary retention and the increased risk of urinary tract infections (UTIs) remain concerns, especially in elderly or frail patients. Despite these challenges, Botox is a promising option for patients unresponsive to conventional treatments, and alternative delivery methods, such as liposome-encapsulation, are being explored to enhance safety and efficacy [95,96,97].

#### 2.1.5. Botulinum Toxin in Chronic Migraine

Botulinum toxin type A (Botox) is a proven prophylactic treatment for chronic migraine (CM), characterized by headaches on at least 15 days per month, with migraine features on 8 or more days. Clinical trials, including the PREEMPT studies, demonstrated its effectiveness in significantly reducing headache days, pain intensity, and reliance on acute medications while improving the quality of life. Botox is administered via injections into specific head and neck muscle groups, with treatment cycles repeated every 12 weeks. Its mechanism involves blocking neurotransmitters like CGRP and glutamate, reducing pain transmission and neuronal sensitization. While generally well tolerated, common side effects include mild injection-site discomfort and muscle weakness. Botox offers a valuable option for patients with refractory CM, providing substantial relief and an enhanced quality of life with sustained use [98,99]. A review article explores the historical development of botulinum neurotoxin (BoNT) in the context of chronic migraine (CM) treatment and examines the latest clinical evidence supporting the use of onabotulinumtoxinA as a therapeutic option for managing CM [100].

#### 2.1.6. Botulinum Toxin in Strabismus

The Cochrane review on the use of botulinum toxin (BoNT) for strabismus concluded that the evidence supporting its efficacy is of low to very low certainty. In children with esotropia, BoNT may offer outcomes similar to surgery, though slightly less effective in achieving ocular alignment. In adults, it appears less effective than surgery for achieving alignment within 10 prism diopters (PD). For acute sixth nerve palsy, BoNT showed potential for improved ocular alignment compared to observation alone, but the results were not statistically significant. Combining BoNT with surgery provided very uncertain benefits compared to surgery alone. Adverse effects, including temporary ptosis and vertical deviation, were common but reversible. The review highlights the need for high-quality trials to clarify BoNT’s clinical and cost-effectiveness in different strabismus cases [101]. Another review recently published in 2023 [102] found that botulinum toxin therapy for strabismus shows mixed results compared to surgery. Surgery demonstrated a higher likelihood of improving or correcting strabismus (risk ratio 0.72, low-certainty evidence), particularly in achieving proper alignment within six months. However, botulinum toxin may not significantly differ from surgery in achieving binocular single vision or stereopsis. Non-serious adverse events, such as transient ptosis and vertical deviation, were more common with botulinum toxin, but no serious adverse events were reported. The conclusion emphasizes the limited and low-certainty evidence available, highlighting the need for high-quality trials to assess long-term outcomes, the quality of life, and cost-effectiveness, which is consistent with [101].

#### 2.1.7. Botulinum Toxin in Primary Axillary Hyperhidrosis

Botulinum toxin (Botox) therapy significantly reduced sweat production and improved the quality of life in patients with axillary hyperhidrosis over one year. Sweat production decreased from 0.81 g to 0.23 g per 15 min, while the Hyperhidrosis Disease Severity Scale (HDSS) scores improved from 3.4 to 1.5. Quality-of-life metrics, such as the Dermatology Life Quality Index (DLQI), improved significantly, with scores dropping from 19.9 to 6.9, and social and environmental quality-of-life domains showing substantial enhancements. These results underscore Botox’s effectiveness in managing both the physical and psychosocial impacts of hyperhidrosis, with sustained benefits and minimal adverse effects, providing a robust case for its use in clinical practice [103]. A recently published randomized controlled trial in 2024 [104] demonstrated that botulinum toxin A (BoNT-A) effectively reduced sweating in patients with primary axillary hyperhidrosis, achieving a 79% median reduction in sweat production at one-year follow-up. At six months, BoNT-A showed superior efficacy compared to baseline, with a 74% reduction in sweat production. Patient-reported outcomes indicated significant improvements in the quality of life and reductions in sweat severity, with no reports of long-term adverse effects. Procedural pain was reported as mild, and patients noted the treatment’s temporary nature, typically lasting 6–12 months. BoNT-A remains a well-tolerated and effective option for managing axillary hyperhidrosis, particularly for those seeking non-invasive treatment with consistent short-term efficacy [104,105,106,107,108,109,110].

#### 2.1.8. Botulinum Toxin in Blepharospasm

The study on botulinum toxin (BoNT) treatment for blepharospasm demonstrated that BoNT effectively reduced the severity of symptoms and improved the quality of life for patients. Most patients received injections every 13–15 weeks, aligning with standard treatment protocols. Common adverse events included mild and transient eyelid ptosis and muscular weakness, which were expected given the targeted injection sites [111]. The study assessed the long-term efficacy and safety of botulinum toxin A (BoNT-A) for treating blepharospasm (BS) over 12 years. The results showed a significant improvement in symptoms, with an average dose increase from 16 to 18 units per session, a shorter latency period by 0.6 days, and an increase in the therapeutic effect duration by 7 days. Patients experienced a 0.6-point improvement on the Jankovic scale. Side effects, such as ptosis and hematoma, were minimal and occurred in only 6.6% of patients [112]. Further other studies concluded that BoNT-A is a safe and effective long-term treatment for BS, with sustained improvements in clinical outcomes and minimal adverse effects [113,114,115].

#### 2.1.9. Botulinum Toxin in Hemifacial Spasm

Botulinum neurotoxin (BoNT) injections are highly effective and clinically safe for managing hemifacial spasm (HFS), with an 88.2% effectiveness rate in single-arm studies. They significantly alleviate symptoms, improve the quality of life, and reduce anxiety and depression levels, although the effects are temporary, requiring re-injections approximately every 20 weeks. The most common adverse reaction is ptosis, which is mild and reversible, with no long-term safety concerns reported. However, the studies revealed considerable variability in outcomes due to differences in dosage, injection sites, and follow-up durations, emphasizing the need for standardized treatment protocols. Despite these limitations, BoNT remains a preferred non-surgical option, providing substantial relief and improved mental health for HFS patients [116,117].

### 2.2. Botulinum Toxins and COVID

The case report discusses a 41-year-old woman who developed hypersensitivity following a cosmetic botulinum toxin (Botox) injection during a concurrent COVID-19 infection. Four hours after the injection, she experienced periorbital swelling, local pain, pruritus, and red plaques despite having undergone Botox procedures twice before without complications. The hypersensitivity reaction was likely exacerbated by her COVID-19 infection as the virus is known to trigger heightened immune responses, particularly involving neutrophils and the complement system, which can lead to abnormal skin reactions. This case highlights the need for caution when administering Botox during active COVID-19 infection as the immune system’s hyperactivity may increase the likelihood of adverse reactions. The patient was successfully treated with antihistamines and topical corticosteroids, leading to a full recovery within five days. The report underlines the growing number of similar cases during the pandemic, where COVID-19 infection or recent vaccination has been linked to hypersensitivity reactions following Botox injections. Dermatologists and cosmetic practitioners are urged to be vigilant and consider the increased risks of such treatments in the context of COVID-19 [118].

The study [119] investigates the effects of the BNT162b2 mRNA vaccine on the outcomes of botulinum toxin type A (BoNT-A) injections. The study followed 45 patients who underwent BoNT-A treatments for aesthetic purposes and had completed two doses of the COVID-19 vaccine. The findings revealed that the interval between BoNT-A injections significantly decreased after vaccination, suggesting a reduction in the efficacy of BoNT-A as patients required more frequent treatments to maintain the desired effects. However, no severe adverse reactions or hypersensitivity were observed post-vaccination, indicating that the safety profile of BoNT-A remained stable. The exact mechanism behind the reduced efficacy is not yet clear, but it is hypothesized that the immune response triggered by the vaccine may affect BoNT-A ’s effectiveness. Despite this, the study emphasizes that the benefits of COVID-19 vaccination far outweigh the need for more frequent cosmetic treatments, and vaccination should not be discouraged. Further research is needed to understand the underlying causes of this reduced efficacy and to explore the impact of actual COVID-19 infection on BoNT-A injections [119].

Botulinum toxin A (BoNT-A) is recognized as a highly effective and safe treatment for managing conditions such as dystonia and spasticity, with precise injection intervals being critical to maintain its therapeutic effect. However, during the COVID-19 pandemic, outpatient clinics in Austria were shut down from November to December 2020, leading to significant delays in treatment schedules. A survey conducted between April and June 2021 with 31 patients from the BoNT outpatient clinic in Horn, Austria, assessed the impact of these delays on patients’ symptoms, physical functioning, and the quality of life (QoL). The patients, with a mean age of 63.7 years, had experienced delays of at least two weeks, with a median delay of 10 weeks. The findings revealed that 94% of patients with spasticity reported worsening muscle cramps, while 91% of dystonia patients experienced increased muscle contractions. Pain was reported by 9% of those with dystonia and 59% of those with spasticity. Overall, 79% of patients reported functional deterioration, and the average reduction in QoL was 61%. The decline in QoL was strongly correlated with the subjective improvement that patients typically experienced after receiving their BoNT-A injections. Additionally, 76% of patients stressed the importance of long-term access to BoNT-A therapy, and 79% expressed that their patient rights were not respected during the shutdowns. This survey underscored the critical importance of uninterrupted BoNT-A treatment for symptom relief, functional outcomes, and overall QoL in patients with movement disorders, even in the face of challenges such as the COVID-19 pandemic [120].

The study [121] explores how the pandemic affected dystonia and hemifacial spasm patients reliant on regular botulinum toxin (BoNT) treatments. Due to healthcare shutdowns, many patients faced treatment delays averaging 8.5 months, significantly worsening their symptoms. The majority of patients, predominantly women with conditions like torticollis and blepharospasm, reported increased muscle contractions and pain. Despite 94% of patients wanting to continue their regular treatments, many lacked access to alternative clinics during the pandemic, leading some to try alternative therapies like acupuncture. Although patients’ physical symptoms deteriorated, with 74% reporting functional decline, there was no significant correlation between treatment delays and overall health scores. On average, patients rated their health at 61.1 on the EQ-5D scale, with 60% scoring their health above 50. This result suggests that while patients experienced worsened symptoms, their immediate perceptions of health may have been influenced by the prospect of receiving BoNT treatment again and might not fully capture the long-term impact of treatment delays [121].

The article [122] addresses the significant impact of the pandemic on medical facilities, particularly on botulinum toxin (BoNT) services for treating neuromuscular conditions like spasticity, dystonia, and sialorrhea. During the early months of the pandemic, many BoNT clinics were either closed or significantly restricted, leading to substantial delays in treatment for patients who rely on regular injections to manage their conditions. Surveys from Germany and Italy revealed that these delays caused an increase in symptoms, including muscle cramps, pain, and a decrease in the quality of life. More than 80% of patients felt their health deteriorated and reported that their rights as patients were neglected due to the shutdowns. The article emphasizes that BoNT services should be deemed essential, even during a global crisis, as delays in treatment can lead to serious health complications for patients.

The recommendations outlined in the article focus on mitigating these issues by improving patient care through telemedicine and other innovative approaches. Telemedicine, for instance, has emerged as a useful tool for assessing patients’ needs and symptoms remotely, allowing for more flexible treatment schedules while minimizing the risk of SARS-CoV-2 exposure. The authors suggest that remote assessments could optimize treatment intervals and help maintain BoNT efficacy, though they also acknowledge that face-to-face assessments are crucial for accurate muscle tone evaluation. Another recommendation is to explore the possibility of home-based or community-based treatments to reduce patient exposure in hospital settings. Additionally, the article underscores the importance of ongoing training for physicians, particularly with telemedicine tools and remote assessments, which could enhance service delivery even beyond the pandemic. Ultimately, the paper sees the COVID-19 crisis as an opportunity to modernize BoNT services, ensuring better patient-centered care and continuity of treatment in the future [122].

### 2.3. Botulinum Toxin and Immunogenicity

Immunogenicity in botulinum toxin (BoNT) therapy involves the formation of neutralizing antibodies (NAbs) that reduce the toxin’s therapeutic efficacy. This risk is higher with frequent injections, high doses, and formulations containing accessory proteins. Patients with NAbs may experience reduced or no response to treatment. Strategies to mitigate immunogenicity include optimizing dosing regimens, extending injection intervals, and using less immunogenic formulations or alternate serotypes. These approaches aim to sustain the effectiveness of BoNT therapy over time [123].

Therapeutic proteins, like onabotulinumtoxin A, carry a risk of triggering an immune response. The detection of antibodies depends on the accuracy and sensitivity of the testing methods used. Several factors can affect the results, including the assay technique, how samples are handled, the timing of sample collection, concurrent medications, and the patient’s health condition. Therefore, comparing the antibody formation rates across different studies or products may not provide reliable conclusions.

A review study focused on how repeated injections for chronic conditions can lead to the development of neutralizing antibodies. These antibodies can cause secondary treatment failure in some patients, although not all patients with antibodies become non-responsive. Differences in botulinum toxin preparations may influence immunogenicity outcomes [124,125].

The immunogenicity rate for all type A botulinum neurotoxins is generally low, while type B serotype formulations tend to be more immunogenic. However, variations in assay sensitivity, sample handling, and underlying disease can affect the reported rates, making product comparisons challenging. Treatment failure and non-response to botulinum neurotoxin are often caused by factors beyond neutralizing antibodies. To minimize the risk of antibody development, clinical strategies focus on using the lowest effective dose and extending the time between injections, reducing the potential for immunogenicity and secondary treatment failure [126].

The review in 2024 highlights that immunogenicity in botulinum toxin A (BoNT-A) therapy is driven by the formation of neutralizing antibodies (NAbs), which reduce therapeutic efficacy over time. Key factors influencing NAb development include high doses, frequent injections, and product-specific attributes like accessory proteins. Effective management strategies involve optimizing dosing regimens, extending treatment intervals, and selecting formulations with lower immunogenic potential to sustain long-term treatment success [127,128,129].

### 2.4. Adverse Events of Botulinum Toxin Treatment

Adverse events due to therapeutic and cosmetic injection of BoNT reported to the FDA include respiratory problems, dysphagia, seizure, flulike syndrome, facial and other muscle weakness, ptosis, and skin and injection site reactions [130]. Of the 406 reports related to therapeutic use, 217 met the FDA’s definition of serious, with 28 deaths and 17 seizures reported. Clinical characteristics submitted to the FDA for therapeutic cases differed from those of cosmetic BoNT cases, which were usually less serious. Most of the adverse effects were linked to the local tissue diffusion of BoNT. Careful attention to drug dose, dilution, handling, storage, and the site of injection are required for optimal treatment outcome and to minimize adverse effects.

A retrospective study examining 1980 injection episodes of botulinum toxin A (BoNT-A) therapy in children with cerebral palsy revealed both systemic and respiratory complications. Bladder or bowel incontinence occurred in 1% of cases and typically resolved within 1 to 6 weeks, indicating potential systemic spread of the toxin, particularly in children with higher Gross Motor Function Classification System (GMFCS) levels. Respiratory complications, including unplanned hospital admissions (1.3%) and upper respiratory tract infections (0.5%), were more frequent in children with severe conditions, such as those at GMFCS levels IV and V. One death due to respiratory complications was reported in a child at GMFCS level V. Higher doses of BoNT-A were associated with increased risks of adverse events, emphasizing the need for careful dose adjustments, especially for children with pre-existing respiratory illnesses. The study recommends considering alternatives to mask anesthesia for high-risk groups and highlights the importance of vigilance in managing children with severe cerebral palsy despite the overall low incidence of serious adverse effects [131,132,133,134].

The article [133] investigates the incidence of adverse events following botulinum toxin A (BoNT-A) injections in children with cerebral palsy (CP). The study, which analyzed 2219 injection episodes in 591 children, focused on understanding how the severity of CP—categorized by the Gross Motor Function Classification System (GMFCS)—influences the occurrence of adverse events. Adverse events were reported during the procedure (6% of injection episodes) and at follow-up (22%). The study found that children with more severe CP (GMFCS levels IV and V) experienced significantly higher rates of systemic adverse events, including lower respiratory tract illnesses and generalized weakness, compared to those with milder forms of CP (GMFCS levels I-III). Notably, children at GMFCS levels IV and V had an increased incidence rate ratio (IRR) of systemic events, with IRRs of 3.92 and 7.37, respectively. Despite the relatively high incidence of adverse events, most were mild and self-limiting. The study emphasizes the need for careful consideration of the risks and benefits of BoNT-A treatment in children with severe CP, highlighting the complex relationship between CP severity and adverse outcomes [133].

The paper [135] extensively discusses various adverse effects associated with the cosmetic use of botulinum toxin type A (BoNT-A). The most common adverse effects are mild and transient, primarily including pain, bruising, and hematomas at the injection sites. Specifically, eyelid and brow ptosis are among the significant adverse events, particularly in the periocular region. The likelihood of these effects increases when improper injection techniques or excessive dosages are used. Other adverse effects, such as headaches, flu-like symptoms, and generalized weakness, have been reported in rare cases. In some instances, BoNT-A diffusion can lead to systemic effects, like respiratory issues or muscle weakness at sites distant from the injection area. The paper emphasizes that the majority of these adverse events are preventable with proper injection techniques, precise dosage, and a comprehensive understanding of the facial anatomy. Additionally, patients with certain neuromuscular disorders or those taking medications like aminoglycosides are at a higher risk of more serious adverse reactions. Nonetheless, the review concludes that with careful patient selection and skilled administration, BoNT-A remains a safe and effective treatment for cosmetic purposes [135].

The paper [136] compares the adverse events of trigonal and extratrigonal injections of botulinum toxin A (BoNT-A) for treating detrusor overactivity (DO). The analysis of six studies with 258 patients revealed that there were no significant differences in the incidence of adverse events between the two techniques. Acute urinary retention (AUR) occurred in 4.2% of trigonal cases compared to 3.7% in extratrigonal cases. Similarly, high post-void residual (PVR) was slightly more common in the trigonal group (25.8%) than in the extratrigonal group (22.2%), but these differences were not statistically significant. Other adverse events such as urinary tract infections (UTIs), hematuria, and postoperative muscle weakness also showed no significant differences between the two groups. Overall, the study concluded that both trigonal and extratrigonal BoNT-A injections are similarly safe, with a low incidence of adverse events, making either technique viable depending on the clinical situation [136].

The review of adverse event (AE) reports associated with botulinum toxin type A (BoNT-A) use, spanning December 1989 to May 2003, analyzed 1437 AE reports 406 following therapeutic use and 1031 following cosmetic use. Among therapeutic users, 217 serious AEs were identified, including all 28 reported deaths, indicating a higher risk for serious events, potentially due to higher doses and complex underlying conditions. For cosmetic use, 36 serious AEs were reported, none involving death, and 30 of these were already recognized in the FDA-approved label. Among non-serious cosmetic AEs (995 reports), the most common issues were the lack of effect (63%), injection site reactions (19%), and ptosis (11%). The study concluded that serious AEs were more frequently reported for therapeutic use, while cosmetic use presented fewer and mostly expected AEs. Limitations include incomplete reporting by clinicians and deviations from FDA-approved guidelines for drug administration [137].

## 3. Discussion

Botulinum toxin (BoNT), particularly BoNT-A, has revolutionized the management of neurological, urological, and dermatological conditions, including its use in scar treatment [138], pain management [139], and cosmetic applications [140]. Its efficacy in treating dystonia, spasticity, chronic pain, and hyperhidrosis is well established, offering significant symptom relief and an improved quality of life. Additionally, BoNT-A is the most widely performed non-surgical aesthetic procedure worldwide, providing millions of patients with effective cosmetic enhancements [141].

Moreover, BoNT is considered safe for aesthetic use in cancer patients [141]. However, despite its widespread clinical application, concerns regarding immunogenicity, secondary treatment failure, and long-term efficacy remain central to ongoing research [137,138,139,141,142].

A limitation of BoNT therapy is the development of neutralizing antibodies (NAbs), which can lead to secondary treatment failure. Repeated BoNT administration, particularly at high doses, has been associated with an increased risk of NAbs, reducing clinical effectiveness [143,144]. Meta-analyses suggest that dystonia and spasticity patients exhibit the highest NAb incidence, with up to 7.4% of patients developing resistance to treatment. This highlights the necessity for careful dose optimization, appropriate injection intervals, and the use of purified formulations with reduced complexing proteins to mitigate immune responses [125,145,146,147].

Recent innovations in BoNT formulations have aimed to minimize immunogenicity. Traditional BoNT-A preparations, such as onabotulinumtoxinA (Botox^®^) and abobotulinumtoxinA (Dysport^®^), contain complexing proteins, which may act as adjuvants, increasing the probability of NAb formation [125]. In contrast, incobotulinumtoxinA (Xeomin^®^) lacks complexing proteins, reducing the risk of immunogenic responses while maintaining clinical efficacy. However, due to variations in assay sensitivity and study methodologies, the true prevalence of NAbs remains uncertain.

Clinical studies have demonstrated that incobotulinumtoxinA exhibits significantly lower immunogenicity, making it a preferable option for patients requiring long-term treatment [144]. Another limitation influencing BoNT efficacy is the dose selection and injection technique. Studies have emphasized that administering higher BoNT doses may not necessarily improve outcomes and may instead contribute to increased antibody formation [15]. Moreover, refining injection site selection and techniques, such as electromyography (EMG)-guided applications, has been shown to enhance treatment precision, reducing the total dose required while maximizing therapeutic benefits [125,148,149]. Recent research has explored new BoNT serotypes, such as BoNT-X, which demonstrate distinct mechanisms of action and potential therapeutic advantages over traditional BoNT-A formulations. BoNT-X has shown promise in targeting alternative SNARE proteins, potentially expanding its applications to conditions that are resistant to current BoNT-A and BoNT-B treatments [24,150,151]. However, further clinical studies are needed to establish its long-term safety and efficacy.

In terms of safety, while BoNT is generally well tolerated, concerns about its long-distance effects remain a topic of ongoing debate. Some studies provide evidence suggesting that BoNT, particularly BoNT-A, can undergo retrograde transport to the central nervous system (CNS), potentially influencing distant neural pathways beyond the injection site. This is supported by findings demonstrating the cleavage of SNAP-25 in central neurons following peripheral BoNT administration [42,43]. However, other studies challenge the clinical significance of these findings, arguing that the majority of BoNT’s effects remain localized when used at therapeutic doses [44]. Additionally, inconsistencies in study methodologies, variability in toxin serotypes, and differences in experimental models contribute to conflicting conclusions regarding the extent and impact of long-distance effects [45,46]. While some reports suggest systemic spread leading to unintended muscle weakness [47], other studies indicate negligible diffusion beyond the targeted muscles, especially in well-controlled clinical settings [48]. Given these discrepancies, further high-quality research is needed to clarify the mechanisms, risk factors, and clinical implications of long-distance BoNT effects, ensuring a balanced understanding of both its therapeutic potential and safety profile [49].

A further limitation of the existing research is the variability in study designs. Many clinical trials evaluating BoNT’s efficacy in dystonia, pain management, and overactive bladder differ in terms of dosing protocols, injection sites, and patient-selection criteria. For example, while some studies support BoNT as an effective treatment for chronic migraine, others indicate only marginal benefits compared to placebo [50,51]. This discrepancy may be due to heterogeneous patient populations, differences in pain assessment tools, or potential placebo effects that are not well accounted for in some studies [52]. Additionally, publication bias is a concern as studies with positive results are more likely to be published, whereas negative or inconclusive findings may remain underreported [53].

Furthermore, industry-funded studies constitute a significant portion of BoNT research. While this funding is crucial for advancing clinical applications, it may introduce bias in study design, data interpretation, or the reporting of adverse effects. Studies funded by pharmaceutical companies producing BoNT formulations tend to report more favorable efficacy and safety outcomes compared to independent research [54,55].

To enhance the effectiveness of BoNT therapy, future research should prioritize personalized treatment strategies by customizing BoNT administration according to each patient’s response, the risk of antibody development, and disease progression. Additionally, investigating nanoparticle-based BoNT delivery could improve targeting precision while minimizing systemic spread.

## 4. Conclusions

Botulinum toxin (BoNT) has evolved from a lethal neurotoxin to a highly versatile therapeutic agent with broad clinical applications in neurology, pain management, urology, dermatology, and aesthetic medicine. Its ability to selectively inhibit neurotransmitter release by targeting SNARE proteins has enabled significant advancements in treating movement disorders, spasticity, chronic pain, overactive bladder, and hyperhidrosis, among others. Despite its efficacy, challenges such as long-distance effects, immunogenicity, and adverse reactions necessitate ongoing research to optimize its safety profile. The development of novel BoNT serotypes, innovative delivery mechanisms, and improved dosing strategies continue to enhance its therapeutic potential. Future studies should focus on minimizing immunogenicity, refining application techniques, and expanding indications to further solidify BoNT’s role in clinical medicine. BoNT remains a cornerstone in both neurological and aesthetic treatments, underscoring the importance of continuous innovation to maximize benefits while minimizing risks. The balance between efficacy and safety will define its future applications, making ongoing research and clinical advancements essential for its sustained success.

## 5. Methodology

This review is based on a comprehensive literature search conducted in PubMed, Scopus, Web of Science, and the grey literature, focusing on English-language articles published between 1897 and 2025. Specific search terms were used to identify relevant studies, ensuring the coverage of pharmacological applications across multiple specialties. Additional articles were retrieved from reference lists to enhance the comprehensiveness of the review. A total of 155 rigorously selected articles were analyzed, providing an in-depth review of BoNT’s therapeutic use, safety, and efficacy. Additionally, this review critically evaluates the methodological strengths and weaknesses of existing research, highlighting biases, study limitations, and conflicting findings in BoNT literature.

### 5.1. Inclusion and Exclusion Criteria

To ensure the consistency and relevance of this review, specific inclusion and exclusion criteria were applied in selecting studies. The included studies consisted of peer-reviewed articles, clinical trials, and systematic reviews that focused on BoNT’s mechanism of action, therapeutic applications, safety, and efficacy. Only studies that provided quantitative data on treatment outcomes were considered. Additionally, research published in English and sourced from reputable scientific journals, regulatory agencies, and clinical guidelines was prioritized. In contrast, exclusion criteria were set to filter out studies with insufficient data, speculative hypotheses, or case reports with fewer than five patients. Non-peer-reviewed sources, such as conference abstracts and opinion-based editorials, were also excluded. Moreover, studies focusing on non-human models without clinical relevance were not considered. These criteria ensured that only high-quality and clinically significant research was included in the review.

### 5.2. Study Selection Process

The study selection process followed a structured three-step approach to ensure the inclusion of relevant and high-quality studies. First, a title and abstract screening was conducted to remove irrelevant or duplicate studies, ensuring that only potentially relevant research was considered. Next, a full-text assessment was performed, where eligible studies were thoroughly reviewed based on their relevance, methodological quality, and alignment with the study objectives. Finally, studies that met the predefined inclusion criteria were selected for synthesis and analysis, forming the basis of this comprehensive review.

## Figures and Tables

**Table 1:** FDA-approved botulinum toxin.

Year	FDA Approval
12/1989	FDA approval for strabismus, blepharospasm, and hemifacial spasm (used in eye muscle disorders and facial spasms).
12/2000	FDA approval for cervical dystonia (a condition that causes abnormal head positioning and neck pain due to muscle spasms).
4/2002	FDA approval for glabellar lines (frown lines between the eyebrows, for cosmetic use).
7/2004	FDA approval for severe primary axillary hyperhidrosis (excessive underarm sweating).
3/2010	FDA approval for upper limb spasticity in adults (muscle stiffness in the arm and hand muscles).
10/2010	FDA approval for chronic migraine (headaches occurring on 15 or more days a month, lasting 4 h or more per day).
8/2011	FDA approval for a specific form of urinary incontinence (due to neurogenic detrusor overactivity).
1/2013	FDA approval for overactive bladder symptoms in adults (for reducing bladder muscle activity that leads to incontinence).
9/2013	FDA approval for severe lateral canthal lines (crow’s feet, for cosmetic purposes).
1/2016	FDA approval for pediatric patients with upper limb spasticity (muscle stiffness in children’s arms and hands).
10/2019	FDA approval for lower limb spasticity, excluding spasticity caused by cerebral palsy (muscle stiffness in legs).
7/2020	FDA approval for pediatric patients with spasticity (muscle stiffness conditions).
2/2021	FDA approval for pediatric detrusor overactivity associated with neurologic conditions (bladder overactivity in children).
7/2021	FDA approval to include eight new muscles to treat adults with upper limb spasticity (expanding treatment options).

## Data Availability

All data are provided in this review article. No new data were generated or analyzed in this study. The review is based entirely on publicly available literature and referenced sources, which are cited appropriately within the manuscript.

## Abbreviations

The following abbreviations are used in this manuscript

AE	Adverse Event
AUR	Acute Urinary Retention
BoNT	Botulinum Neurotoxin
BTX	Botulinum Toxin
COL-CAP	Classification System for Cervical Dystonia
CGRP	Calcitonin Gene-Related Peptide
CPM	Conditioned Pain Modulation
GMFCS	Gross Motor Function Classification System
MFP	Myofascial Pain (Syndrome)
NTNH	Stands for Non-Toxic Non-Hemagglutinin
NAbs	Neutralizing Antibodies
OAB	Overactive Bladder
PREEMPT	Phase III Research Evaluating Migraine Prophylaxis Therapy
SNARE	Soluble N-ethylmaleimide-sensitive factor Attachment Protein Receptor
RCT	Randomized Controlled Trial
SV2	Synaptic Vesicle Protein 2
TeNT	Tetanus Neurotoxin
UTIs	Urinary Tract Infections
